# The genome sequence of *Synechocystis* sp. PCC 6803 substrain GT‐T and its implications for the evolution of PCC 6803 substrains

**DOI:** 10.1002/2211-5463.13576

**Published:** 2023-02-21

**Authors:** Satu Koskinen, Juha Kurkela, Markéta Linhartová, Taina Tyystjärvi

**Affiliations:** ^1^ Department of Life Sciences/Molecular Plant Biology University of Turku Finland; ^2^ Institute of Microbiology of the Czech Academy of Sciences Třeboň Czech Republic

**Keywords:** chromosome sequence, cyanobacteria, *Synechocystis* sp. PCC 6803

## Abstract

*Synechocystis* sp. PCC 6803 is a model cyanobacterium, glucose‐tolerant substrains of which are commonly used as laboratory strains. In recent years, it has become evident that ‘wild‐type’ strains used in different laboratories show some differences in their phenotypes. We report here the chromosome sequence of our *Synechocystis* sp. PCC 6803 substrain, named substrain GT‐T. The chromosome sequence of GT‐T was compared to those of two other commonly used laboratory substrains, GT‐S and PCC‐M. We identified 11 specific mutations in the GT‐T substrain, whose physiological consequences are discussed. We also provide an update on evolutionary relationships between different *Synechocystis* sp. PCC 6803 substrains.

## Graphical abstract

The chromosome of glucose‐tolerant GT‐T substrain of *Synechocystis* sp. PCC6803 was sequenced. We found 11 GT‐T‐specific mutations (A). The GT‐T substrain is nonmotile like all other glucose‐tolerant substrains because it contains only thin pili and thin pili bundles (B). The frameshift mutation in the glucose transporter gene prevents photoheterotrophic growth (C).

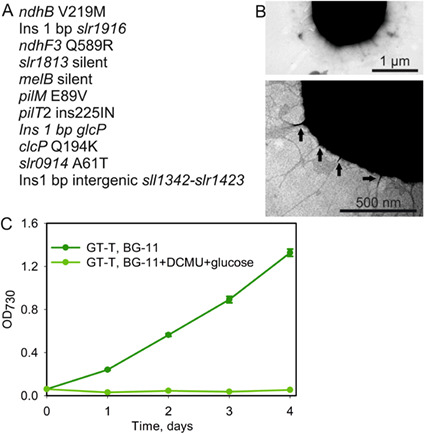

**Abbreviations**
ATCC: American Type Culture Collection
DCMU: 3‐(3,4‐dichlorophenyl)‐1,1‐dimethylurea
PPFD: photosynthetic photon flux density
SNP: single nucleotide polymorphism

The cyanobacterium *Synechocystis* sp. PCC 6803 (hereafter *Synechocystis*) is a unicellular, non‐nitrogen‐fixing, freshwater cyanobacterium that is widely used as a model organism. It was isolated in California in 1968 and deposited to the Pasteur Culture Collection of Cyanobacteria (*Synechocystis* sp. PCC 6803) and to the American Type Culture Collection (ATCC 27184) [1].

The popularity of *Synechocystis* as a model organism is based on a few advantages. The complete chromosome sequence of *Synechocystis* was available already in 1996 [2]. Construction of mutant strains is easy in this naturally competent cyanobacterium [3]. The original PCC 6803 or ATCC 27184 strains do not tolerate glucose in the light, but later glucose‐tolerant substrains allowing photoheterotrophic, mixotrophic, and heterotrophic growth have been isolated [4, 5, 6, 7]. A large variety of biophysical, biochemical, and molecular biology techniques have been optimized to *Synechocystis*.

The 1996 sequenced strain was a substrain Kazusa (GT‐Kazusa). GT‐Kazusa is a derivative of the glucose‐tolerant Williams strain [4]. Phenotypic variations and some known sequence differences between *Synechocystis* substrains led to the sequencing of the substrain GT‐S (substrain from Sato's laboratory in Tokyo) in 2011 [8]. GT‐S originated from the same laboratory strain as GT‐Kazusa. Due to their close history, it was assumed the GT‐S and GT‐Kazusa would have identical sequences. However, a comparison of the GT‐S sequence and re‐sequenced frozen DNA samples of GT‐Kazusa revealed that these strains contain a few differences [8].

Thereafter, more *Synechocystis* substrains have been sequenced: GT‐I, PCC‐P and PCC‐N [9], PCC‐M [10], GT‐O1 and GT‐O2 [11], GT‐G [7], and GT‐P and GT‐W [12]. Based on sequence data, *Synechocystis* substrains can be divided into two main clades, the GT clade comprise nonmotile, glucose‐tolerant descendants of the ATCC 27184 strain, whereas PCC strains are descendants of *Synechocystis* sp. PCC 6803 strain in the Pastor Culture Collection of Cyanobacteria. Some sequence differences between GT and PCC strains are common to all sequenced substrains, but on the top of these common features, all sequenced strains contain numerous substrain‐specific mutations.

To allow full comparison of the results obtained with different substrains and mutants constructed using them as host strains, we have now sequenced our substrain, named as GT‐T.

## Materials and methods

### *Synechocystis* sp. PCC 6803 substrains and growth conditions

The GT‐T substrain of *Synechocystis* sp. PCC 6803 was brought to Turku from Christer Jansson's laboratory in the early 90s [13, 14]. To Stockholm, Christer Jansson brought it from McIntosh's laboratory in the late 80s [15]. The AR mutant strain, derived from the glucose‐tolerant McIntosh's strain, contains interrupted *psbA1* and *psbA3* genes and the antibiotic resistance cassette after the *psbA2* gene [14]. Substrain Nishiyama is used as a control strain in Nishiyama's laboratory [16] and the substrain GT‐P is from Nixon's laboratory [12].

Cells were grown in our standard growth conditions. The BG‐11 medium was supplemented with Hepes‐NaOH pH 7.5 and cells were grown at 32 °C in ambient air. Thirty‐mL cell cultures were grown in 100‐mL Erlenmeyer flasks under constant illumination with the photosynthetic photon flux density (PPFD) of 40 μmol m^−2^ s^−1^. The light source was a mixture of fluorescence tubes, light colors 840 and 865 [Osram (Munich, Germany)/Philips (Amsterdam, The Netherlands)]. For photoheterotrophic conditions, cultures were supplemented with 5 mm glucose and 10 μm 3‐(3,4‐dichlorophenyl)‐1,1‐dimethylurea (DCMU). Growth was followed by measuring OD_730_ once a day. Dense cultures were diluted (OD_730_ did not exceed 0.4 in measurements) and dilutions were taken into account when the results were calculated.

### DNA sequencing

Genomic DNA was isolated using the phenol extraction method [4]. Sequencing was done by Eurofins Genomics Europe Sequencing Gmbh (Konstanz, Germany) with NovaSeq 6000 platform and 150 bp paired‐end configuration. For library preparation, TruSeq Adapter sequences were utilized using a self‐validated and established protocol of Eurofins Genomics, based on NEBNext Ultra II Directional DNA Library Prep Kit for Illumina. Default parameters were used in the following analysis tools. The quality control for the 3,651,578 Illumina raw reads was done with FastQC v.0.11.3 [17].

### Mapping of DNA reads to the chromosomes of *Synechocystis* sp. PCC 6803 substrains GT‐S and PCC‐M

Reads (30 × sequence coverage) were mapped to the reference genome sequence of *Synechocystis* sp. PCC 6803 substrain GT‐S (https://www.ncbi.nlm.nih.gov/nuccore/NC_017277) using BWA‐MEM aligner v.0.7.12‐r1039 [18] using the following parameter setting: Minimum seed length 19, Maximum gap length 100, Match score 1, Mismatch penalty 4, Gap opening penalty 6, Gap extension penalty 1, Penalty for end clipping 5. GT‐S reference genome was modified by removing a transposase gene sequence (nucleotide position 2047218 to 2048400), as only GT‐Kazusa and GT‐S contain this transposase [19]. Variant calling was done with SAMtools v.1.2 + htslib‐1.2.1 [20] using the following setting: Minimum mapping quality for an alignment to be used 0, Minimum base quality for a base to be considered 20, Output per sample read depth yes, Output per sample number of high‐quality nonreference bases yes. All data analysis steps were performed with Chipster v4 platform [21]. The chromosome was annotated using NCBI Prokaryotic Genome Annotation Pipeline (PGAP) with the best‐place reference protein set and GeneMarkS‐2+ v6.1 [22, 23, 24]. In addition, the reads of GT‐T were mapped to the genome sequence of the substrain PCC‐M (https://www.ncbi.nlm.nih.gov/nuccore/CP003265).

### Negative staining of pilus structures

Cells (1 mL, OD_730_ = 0.4) were pelleted at 3000 ***g*** for 5 min and fixed in 1 mL of 1% glutaraldehyde, pelleted again, and resuspended in fresh BG‐11 medium. Cells were allowed to adhere to formvar‐coated copper grids (300 mesh, Agar Scientific, Stansted, UK) for 5 min. Then, the grid was drained and negatively stained for 1 min with 3% aqueous uranyl acetate and examined in a JEOL JEM‐1010 transmission electron microscope equipped with a CCD Sis MegaView III (Olympus, Tokyo, Japan) at Laboratory of electron microscopy (Biology centre CAS, České Budějovice, Czech Republic).

### Determination of chlorophyll

Cells from 1 mL of culture (OD_730_ = 0.6) were collected by centrifugation at 9400 ***g*** for 1 min. The pellet was resuspended into 1 mL of 100% methanol and incubated for 30 min. After centrifugation at 13,500 ***g*** for 5 min, OD_665_ was measured and chlorophyll content was calculated using the extinction coefficient as in [25].

### Immunological detection of the ω subunit of RNA polymerase

Cells were grown in the standard growth conditions (30 mL, OD_730_ = 0.6) and collected by centrifugation at 4000 ***g*** for 5 min at 4 °C, and proteins were isolated as in [26]. Protein concentration was determined with the DC protein assay kit (Bio‐Rad, Herculaes, CA, USA). Samples containing 25 μg of total proteins were solubilized with Laemmli's solubilization buffer at 75 °C for 10 min and separated by Next Gel SDS/PAGE according to the manufacturer's instructions (Amresco, Radnor, PA, USA). Thereafter, proteins were transferred to Immobilon‐P membrane (Millipore, Burlington, MA, USA) and the α and ω subunits of the RNA polymerase (Agrisera, Vännäs, Sweden; custom antibodies described in [19]), and the large subunit of Rubisco (Agrisera, AS03037) was immunodetected with the specific antibodies as in [19]. The goat anti‐rabbit IgG (H + L) alkaline phosphatase conjugate (Zymed, ThermoFisher, Waltham, MA, USA) and CDP‐star chemiluminescence Reagent (PerkinElmer, Waltham, MA, USA) were used for the signal detection. Immunoblots were quantified using Epson perfection V600 photo scanner (Epson, Suwa, Nagano, Japan) and image j program [27].

## Results

The DNA from the GT‐T substrain was isolated using the phenol extraction method [4] and sent for sequencing to Eurofins Genomics Europe Sequencing Gmbh. The genome of the GT‐S substrain [9] was selected as a reference genome and reads from the GT‐T genome were mapped to the GT‐S genome. The length of the GT‐T chromosome was 3,569,929 bp with an overall GC content of 47.74%. Mapping reads of GT‐T genome to the GT‐S reference genome revealed 12 differences between the chromosomes of GT‐T and GT‐S (Table 1). The GT‐T chromosome contains one large 1183 bp long deletion, one 6 nt‐long insertion, 3 one nucleotide insertions, and seven single nucleotide substitution mutations (Table 1), altogether making the chromosome of GT‐T 1174 bp shorter than that of GT‐S.

**Table 1:** Location and effects of single nucleotide changes and indels found in the comparison of the nucleotide sequence of the GT‐T substrain to that of the GT‐S substrain in the database.

Position	GT‐S	GT‐T	AA change	Locus	Gene	Function
157961	C	T	Val219Met	*sll0223*	*ndhB*	NAD(P)H‐quinone oxidoreductase subunit 2
619782	–	T	frameshift S56	*slr1916*		Esterase
938475	T	C	Gln589Arg	*sll1732*	*ndhF3*	NAD(P)H‐quinone oxidoreductase subunit F
1290831	G	A	Silent	*slr1813*		Hypothetical protein
1840410	G	A	Silent	*sll1374*	*melB*	
1864740	A	T	Glu89Val	*slr1274*	*pilM*	Type IV pilus assembly protein
2047228		Del 1183 bp		*slr1635*		Transposase
2044508	–	ATCAAC	Insertion Ile225Asn226	*sll1533*	*pilT2*	Pilus retraction protein
2398167	–	C	frameshift G28	*sll0771*	*glcP*	Glucose transporter
2465926	G	T	Gln194Lys	*sll0020*	*clcP*	Clp protease ATP binding subunit
2786264	G	A	Ala61Thr	*slr0914*		Hypothetical protein
3276804	–	A				Intergenic between ORFs *sll1342‐slr1423*

**Fig. 1:**
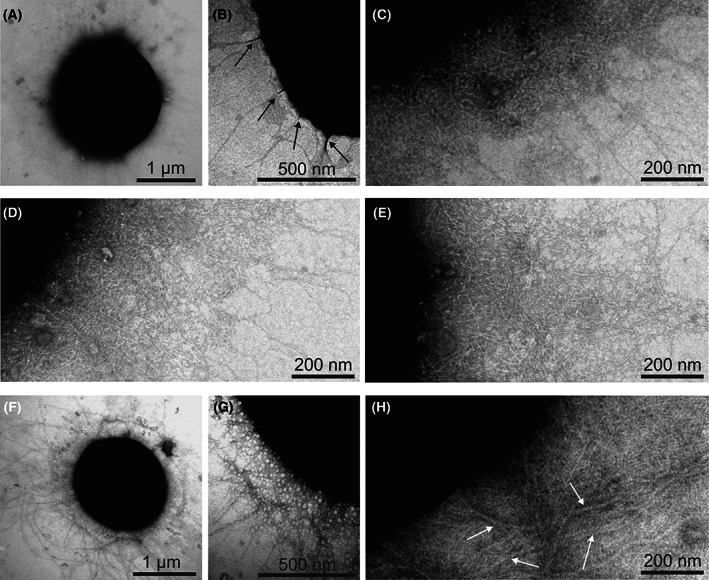
Pili on the surface of *Synechocystis* sp. PCC 6803 nonmotile and motile substrains. Transmission electron microscopy micrographs of nonmotile GT‐T substrain (A–C), nonmotile GT‐W substrain (D, E), or motile PCC‐M substrain (F–H) cells that were negatively stained with 3% aqueous uranyl acetate. Thick pili are indicated with white arrows. Bundles of thin pili are indicated with black arrows.

The long deletion of the GT‐T chromosome comprises transposase *slr1635* (Table 1) that is only found in the substrains GT‐S and Kazusa [9, 10, 11, 19]. The 6 bp long insertion of the GT‐T chromosome locates in the *pilT2* gene causing Ile Asn duplication after amino acid 224 in the PilT2 protein (Table 1). PilT2 is homologous to other conserved PilT motor proteins functioning in the depolymerization process of type IV pili [28]. The *pilM* gene contained the A to T substitution, leading to a Glu89Val mutation in the PilM protein. The PilM protein is suggested to be part of the plasma membrane type IV pili core complex [29]. To figure out if mutations in *pil* genes cause defects in pilus structures, pilus structures were studied by transmission electron microscopy of negatively stained GT‐T, GT‐W, and PCC‐M cells. Representative electron micrographs are shown in Fig. 1. The pili structures of the GT‐T (Fig. 1A–C) closely resemble those of other nonmotile, glucose‐tolerant substrains GT‐W (Fig. 1D,E) or GT‐P [30] but differ from the motile PCC‐M substrain (Fig. 1F,H; for motile strains see also [29, 31]). The GT‐T, GT‐W, and GT‐P substrains display thin pili and thin pili bundles but lack typical type IV thick pili in contrast to the motile substrains.

**Fig. 2:**
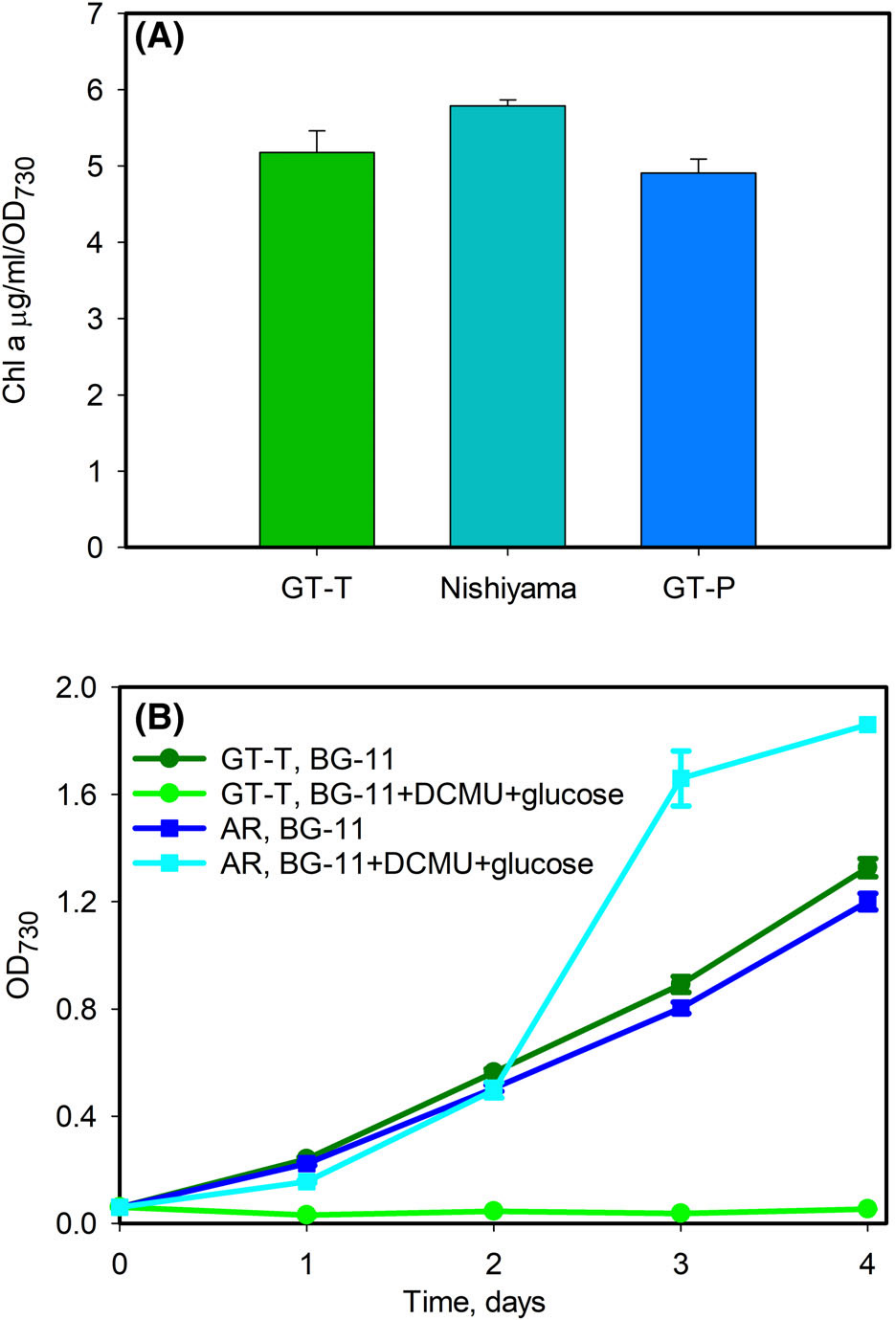
The properties of *Synechocystis* sp. PCC 6803 substrain GT‐T. (A) Chlorophyll a content of substrains GT‐T, GT‐P, and a glucose‐tolerant substrain from Nishiyama's laboratory. (B) Autotrophic and photoheterotrophic growth of the GT‐T substrain and the AR mutant strains (interrupted *psbA1* and *psbA3* genes and the antibiotic resistance cassette after the *psbA2* gene). For autotrophic conditions, 30‐mL cell cultures were grown in 100‐mL Erlenmeyer flasks in the BG‐11 medium supplemented with Hepes‐NaOH pH 7.5 at 3 °C in ambient air with gentle agitation of 90 rpm. Cells were constantly illuminated, PPFD 40 μmol m^−2^ s^−1^. For photoheterotrophic conditions, cell cultures were supplemented with 5 mm glucose and 10 μm DCMU. The results are the average of three independent biological replicates and the error bars denote SEM.

Three one bp insertions were detected in the GT‐T substrain. The insertion of an extra A nucleotide in position 3276804 located in an intergenic region (Table 1) is unlikely to cause any phenotype, but two other insertions cause frameshift mutations. The first one is located in the *slr1916* gene that encodes a putative esterase (Table 1). This esterase has been shown to have chlorophyll dephytylase activity *in vitro* and Δslr1916 was found to have higher chlorophyll content than the control strain [32]. We compared the chlorophyll content of GT‐T, GT‐P, and Nishiyma strains, and detected no difference between the strains (Fig. 2A). Recently, a protein family consisting of DphA1‐3 proteins with dephytylase activity, was characterized in *Synechoccus elongatus* PCC 7942 [33]. Orthologous *dphA* genes were found in numerous cyanobacteria including *Synechocystis* sp. PCC 6803 [33]. Thus DphA enzymes might compensate the missing Slr1961 in the GT‐T substrain.

The second frameshift mutation is located in the *sll0771* gene that encodes a glucose transporter [34]. To test whether our GT‐T strain is able to use externally added glucose, we grew the GT‐T substrain in photoheterotrophic conditions by blocking PSII activity with 10 μm DCMU and simultaneously supplementing the growth medium with 5 mm glucose. The GT‐T cells were not able to grow photoheterotrophically (Fig. 2B) indicating that, indeed, the detected frameshift mutation prevents the function of the glucose transporter. We also tested a cell culture started from the oldest GT‐T glycerol stock cells we have (prepared in 1999), and also those cells contained a nonfunctional glucose transporter. The mutation in the glucose transporter gene has appeared in Turku in 1990s, as the glucose‐tolerant strain in McIntosh laboratory [5], and A2 and AR mutants constructed on that strain were able to grow photoheterotrophically [14, 15, 35]. We also confirmed that AR cells still grow photoheterotrophically (Fig. 2B).

Two of the seven‐point mutations in the GT‐T strain are located in *ndh* genes (Table 1). The NDH‐1 core protein NdhB [36, 37] contains the Val219Met mutation in GT‐T and the NdhF3 protein Gln589Arg mutation; the NdhF3 is a specific subunit for an NDH‐1 complex functioning in carbon acquisition [36, 37]. Next point mutation cause Gln194Lys change in the ATP binding subunit of the Clp protease. The Clp protease is involved in phycobilisome degradation [38], and in addition, some mutations in the ClpC protein have been shown to increase the thermotolerance of *Synechocystis* cells [39]. However, the degradation of phycobilisomes during nitrogen deficiency occurred normally in GT‐T cells [40], and we have not observed increased thermotolerance either [41, 42] suggesting the normal function of the mutated Clp protease. Two open reading frames *slr1813* and *sll1374* contain one silent mutation each and the hypothetic protein Slr0914 contains Ala61Thr mutation.

**Fig. 3:**
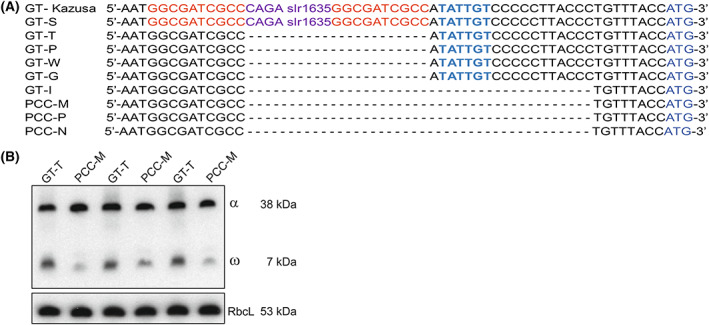
Sequence of the upstream region of the *rpoZ* gene and the content of the α and ω subunits of the RNA polymerase and the large subunit of Rubisco (RbcL) in *Synechocystis* sp. PCC 6803 substrains. (A) Comparison of the upstream region of the *rpoZ* gene in *Synechocystis* substrains. Transposase in violet, target site duplication in red, putative −10 region of the *rpoZ* gene in bold blue, and the initiation codon of the *rpoZ* gene in blue. (B) GT‐T and PCC‐M substrains were grown in standard conditions, total proteins were isolated, solubilized and 25 μg of proteins were separated by Next gel SDS/PAGE. The ω and α subunits of RNA polymerase and the large subunit of Rubisco (RbcL) were immunodetected by Western blotting using specific antibodies. Three independent biological replicates are shown.

When the chromosome sequence of GT‐T was compared to that of PCC‐M, 29 variations were detected between the chromosomes (Table 2). Eleven of those variations were the same as detected in the comparison of GT‐T and GT‐S suggesting that those are specific to GT‐T. Eight of the variations are typical for all PCC substrains, and 9 are specific for PCC‐M (Table 2). Both GT‐T and PCC‐M are missing transposon *slr1635*, but only PCC‐M is missing adjacent 18 bp, just eight nucleotides upstream of the *rpoZ* gene (Fig. 3A). These 18 nucleotides are also missing in PCC‐P, PCC‐N, and GT‐I but not in other GT strains (Fig. 3A). A putative promoter region of the *rpoZ* gene that encodes the nonessential ω subunit of RNA polymerase [19] locates within this area, and therefore we measured the amount of the ω protein in PCC‐M. PCC‐M cells are able to produce the ω subunit, although the amount has decreased circa 60% compared to that in GT‐T (Fig. 3B). The amounts of the α subunit of RNA polymerase and the large subunit of Rubisco (RbcL) were measured as control proteins. Similar amounts of the α subunit of RNA polymerase and RbcL were detected in GT‐T and PCC‐M substrains (Fig. 3B).

**Table 2:** Location and effects of single nucleotide changes and indels found in the comparison of the nucleotide sequence of GT‐T to that of PCC‐M in the database. Clade indicates changes found between GT‐T and all PCC strains (PCC), GT‐T‐specific changes (T), and PCC‐M‐specific changes (M).

Position	Clade	PCC‐M	GT‐T	AA change	Locus	Gene	Function
144507	M	G	A	Silent	*slr0242*	*bcp*	Bacterioferritin comigratory protein
157961	T	C	T	Val219Met	*sll0223*	*ndhB*	NAD(P)H‐quinone oxidoreductase subunit 2
489109	M	C	T	Ser628Leu	*slr1609*		Long‐chain‐fatty‐acid CoA ligase
619284	T	–	T	frameshift	*slr1916*		Esterase
730868	PCC	–	A	frameshift	*sll1574*	*SpkA*	
781267		PCC		Del 154 bp			
831302	M	T	C				Intergenic and *slr2031*
847733	M	A	G	Asn2Ser	*slr1898*	*argB*	N‐acetylglutamate kinase
938130	T	T	C	Gln589Arg	*sll1732*	*ndhF3*	NAD(P)H‐quinone oxidoreductase subunit F
1070494	M	A	T	Lys298Asn	*sll1359*		Hypothetical protein
1203086	PCC	A	G	Tyr95Cys	*slr1865*		Hypothetical protein
1423938	PCC	–	A	frameshift	*sll1951*	*hlyA*	Haemolysin
1290484	T	G	A	Silent	*slr1813*		Hypothetical protein
1810888	PCC	T	C	Val233Ala	*slr1983*		Response regulator
1840062	T	G	A	Silent	*sll1374*	*melB*	
1864392	T	A	T	Glu89Val	*slr1274*	*pilM*	Type IV pilus assembly protein
2044160	T	–	ATCAAC	Ile Asn insertion	*sll1533*	*pilT2*	Pilus retraction protein
2046851	PCC	–	GGGTAAGGGGGACAATAT		intergenic		
2397801	T	–	C	frameshift	*sll0771*	*glcP*	Glucose transporter
2397991	M	A	C				intergenic
2465560	T	G	T	Gln194Lys	*sll0020*	*clpC*	Clp protease ATP binding subunit
2518280	PCC	C	T	Ser928Phe	*slr0222*	*hik25*	Hybrid sensory kinase
2785898	T	G	A	Ala63Thr	*slr0914*		Hypothetical protein
3011933	PCC	C	T	Silent	*slr0302*		PleD‐like protein
3095975	PCC	C	T	Arg103Cys	*ssr1176*		Transposase
3276439	T	–	A				Intergenic
3364288	M	–	A	frameshift	*sll1496*		Mannose‐1‐phosphate guanyltransferase
3369206	M	A	T	Lys239Met	*slr1564*	*sigF*	RNA polymerase SigF sigma factor
3419464	M	T	C	Leu118Pro	*slr0753*		P protein

## Discussion

**Fig. 4:**
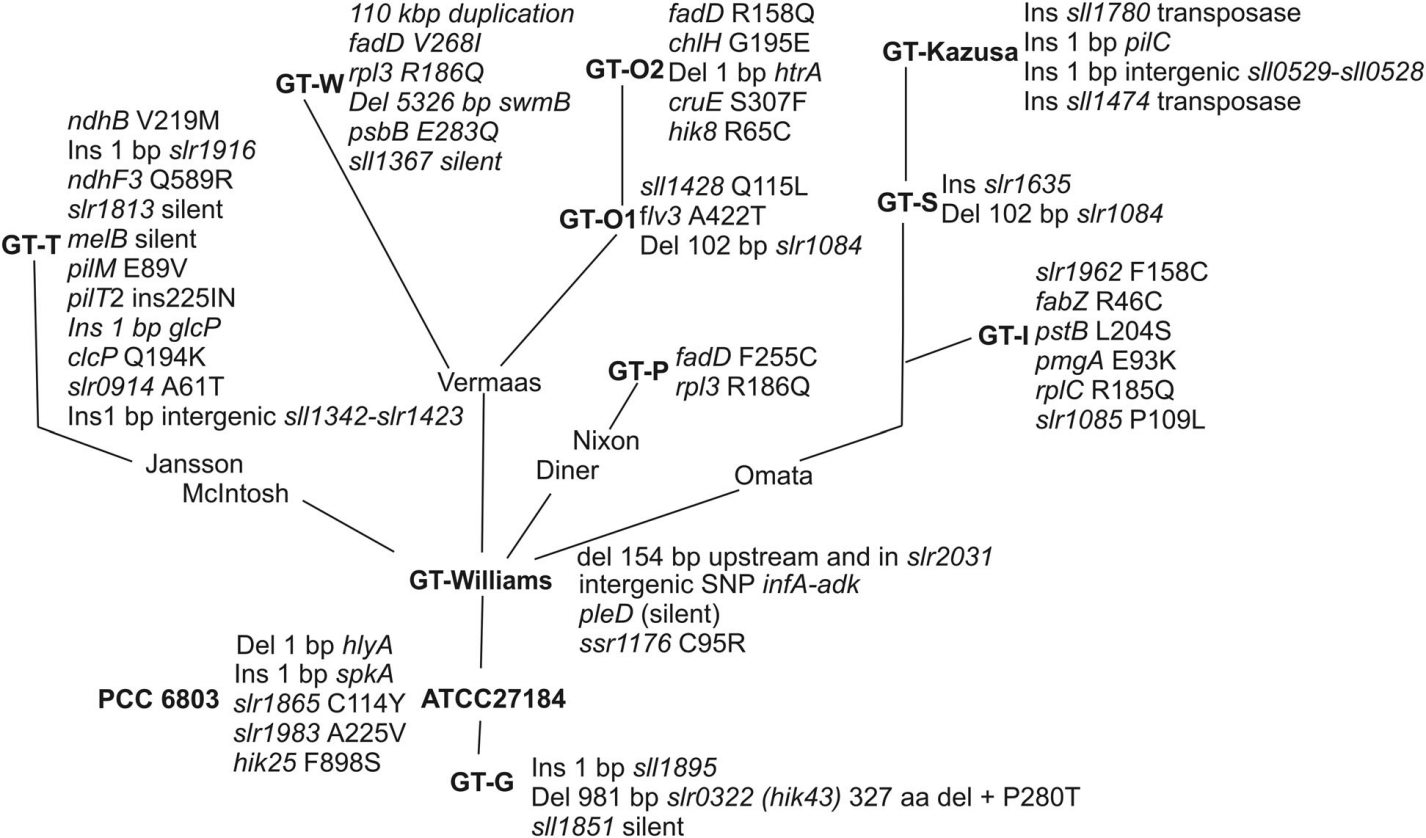
Substrain‐specific mutations in glucose‐tolerant nonmotile *Synecocystis* cells. Isolated *Synechocystis* sp. PCC 6803 was deposited to two culture collections. Nonmotile glucose‐tolerant strains are descendants of the ATCC 27184 strain in the American Type Culture Collection, and the motile PCC strains originate from PCC 6803 strain in the Pastor Culture Collection of Cyanobacteria. Routes of strains to the laboratories, where they were sequenced, are shown according to the information found in the literature. The sequenced strains are GT‐Kazusa and GT‐S (8); GT‐G (7); GT‐I, PCC‐P, and PCC‐N (9); PCC‐M (10); GT‐O1 and GT‐O2 (11); and GT‐P and GT‐W (12); GT‐T (this publication).

The laboratory substrains of *Synechocystis* can be divided into two main clades, the GT clade originates from the ATCC 27184 strain comprising nonmotile glucose‐tolerant strains, the majority of which are descendants of the glucose‐tolerant substrain selected by Williams [4] but include also independently isolated GT‐G strain by Wei's laboratory [7]. The PCC clade containing motile strains originated from the PCC 6803 strain, some PCC substrains being glucose‐sensitive and some glucose‐tolerant [10]. The chromosome sequence of our *Synechocystis* sp. PCC 6803 substrain places it among the GT strains (Fig. 4).

Two frameshift mutations separate all GT and PCC strains. A frameshift mutation in the *spkA* gene destroys the function of the SpkA protein in all GT substrains (Fig. 4). The Spk kinase has been shown to function as a regulator of cell motility [43, 44], and the lack of the SpkA kinase can be considered to be a reason for nonmotile phenotype of GT strains. The frameshift mutation in the *hlyA* gene causes a lack of the full‐length HlyA surface layer protein in PCC strains and might increase the sensitivity of the PCC strains to external stress conditions, although Δsll1951 cells grow well in standard conditions [45].

Three‐point mutations separate all GT and PCC strains (Fig. 4). To figure out which lineage has a mutated version, we compared nucleotide sequences of these three genes in *Synechocystis* substrains to those of close homolog genes in other cyanobacteria. In the *slr1865* gene, the mutation has most probably occurred in the GT lineage as homologous genes in *Synechocystis* sp. CACIAM 05, *Synechocystis* sp. PCC7339, *Synechocystis* sp. PCC7338, *Synechocystis* sp. PCC6714, *Nostocales* HT‐58‐2, and *Halothece* sp. PCC 7818 all contain A nucleotide in position 340 just like the PCC substrains. Whereas in the *hik25* gene, the mutation has occurred in PCC lineage, as *Synechocystis* sp. CACIAM 05, *Synechocystis* sp. PCC7339, *Synechocystis* sp. PCC7338 and *Synechocystis* sp. PCC6714 strains contain T in position 2692, just like the GT substrains. Position 674 in the putative phosphatase gene, *slr1983*, shows variation between the strains being C in GT lineage and in *Synechocystis* sp. CACIAM 05 and *Synechocystis* sp. PCC6714, T in PCC lineage, and A in *Synechocystis* sp. PCC7339 and *Synechocystis* sp. PCC7338.

In addition to common differences between the GT and PCC substrains, all substrains originated from the Williams strain share common mutations (Fig. 4). Intergenic single nucleotide polymorphism (SNP) between *adkA* and *infA* localizes to the putative −10 promoter region of the *infA* gene that encodes a translation initiator factor. However, the physiological consequence of these mutations is unclear, as the *infA* gene belongs to a large gene cluster whose transcript pattern suggests multiple transcription initiation sites within the cluster that can be also transcribed as a large operon [46]. Physiological consequences of the 154 bp deletion in and upstream of the *slr2031* gene also remain to be studied. The *slr2031* gene is a homolog of the *rsbU* gene in *Bacillus subtilis* in which the RsbU phosphatase regulates the function of the stress‐responsive SigB σ factor via the RsbV/RsbW anti‐σ factor/anti‐σ factor antagonist pair [47]. In *Synechocystis*, Δslr2031 cells have been reported to show defects in recovery from nitrogen or sulfur deficiency [48], but in our measurements, the GT‐T substrain enters and recovers from nitrogen deficiency similarly as the PCC‐M substrain [40, 49].

All descendants of the Williams strain [4] have gained additional mutations (Fig. 4). Surprisingly, a completely different set of mutations was discovered when the GT‐W substrain (originating from Vermaas's laboratory) was sequenced in Prague (laboratories of Sobotka and Komenda) and the GT‐O1 and GT‐O2 substrains, also descendants of Vermaas strain, in Otago (laboratory of Eaton‐Rye) [11, 12]. GT‐W contains a large, 110 kb, tandem duplication between the identical transposases *sll0431* and *sll1397* [12]. This large duplication contains 100 genes, and in addition, GT‐W contains four strain‐specific SNPs and a 5326 bp deletion in the *swmB* gene [12]. The phenotype of the GT‐W substrain is highly unique with high carotenoid and low chlorophyll and phycocyanobilin contents of the cells [12]. Interestingly, this phenotype is promoted with glucose supplementation, as the large duplication of the GT‐W chromosome disappears if cells are grown for several months in autotrophic conditions [12].

GT‐T‐specific mutations have consequences on the phenotype of cells. A frameshift mutation has destroyed the glucose transporter, and GT‐T cells do not grow heterotrophically (Fig. 2). We have not realized that earlier, because we have not grown GT‐T cells in photoheterotropic or heterotrophic conditions. When testing *psbA* mutants in 1990s, we often used photoheterotrophic conditions, but in those experiments, we always used the AR strain as a reference strain [50]. Our RNA polymerase mutants have been constructed using GT‐T as a host strain [19, 26, 51, 52], but those mutants have not been tested in photoheterotrophic conditions. Obviously, the GT‐T substrain or mutants constructed using GT‐T as a host strain should not be used in studies utilizing externally added glucose. However, the lack of the functional glucose transporter does not have overall effects on sugar metabolism. An overdose of the SigE σ factor that regulates sugar catabolic genes causes similar transcriptomic responses whether GT‐T [52] or the glucose‐tolerant strain of Tanaka's laboratory is used as a host strain [53].

In the GT‐T substrain, PilM and PilT2 proteins contain mutations on the top of pili regulating kinase SpkA (Table 2). The insertion of Ile225 and Asn226 in the PilT2 protein is located in a conserved domain in the proximity to the crucial Lys212 residue in a Walker A ATP/GT‐P‐binding motif [28] and a manganese‐binding pocket [54], thus potentially affecting the function of the PilT2 protein. The *pilT2* deletion strain, constructed in the motile PCC host strain, remains motile and competent but shows an opposite response to unidirectional light than the host strain [28]. Cells without functional PilM lost both motility and competence [29]. Mutations in *pilT* and *pilM* genes can be assumed to play only minor roles, as pili of GT‐T resemble those of the other nonmotile substrains (Fig. 1, [30]) and GT‐T is competent, just like all other GT substrains, excluding GT‐Kazusa, which has lost competence due to the frameshift mutation in the *pilC* gene [8].

Two *ndh* genes of the GT‐T substrain contain point mutations (Table 1). The NdhB protein is supposed to function both in cyclic electron flow and in CO_2_ acquisition, whereas NdhF3 functions only in carbon acquisition [36, 37]. The GT‐T substrain shows typical responses to high and low CO_2_, indicating that the GT‐T substrain contains functional NDH complexes [55].

It is noteworthy that the movement of transposons is one driving force for substrain‐specific mutations. Furthermore, single nucleotide point mutations (including only few silent mutations) or single nucleotide deletion or insertions seem to concentrate on the coding sequences of genes with known function and show high variation between substrains. It should also be noted that we sequenced DNA isolated from storage cells that are currently in use, whereas the majority of other sequencing projects have been started from old storage cells from the early 1990s. In 1990s, we prepared new storage cells after the old ones had run out, and therefore our current storage cells have been grown for numerous generations after the GT‐T substrain was brought from Stockholm to Turku. After understanding how easily new mutations appear in the genome, we have been more careful in producing storage cells only after a minimal number of cell generations.

Just by comparing sequences, it is not easy to figure out why glucose‐tolerant strains are glucose‐tolerant, as GT and PCC‐M strains do not share mutations that could be directly connected to glucose tolerance. When we grew PCC‐M photoheterotrophically in the presence of DCMU and glucose, cells were growing, but similar fast growth as in the AR strain was not observed. Furthermore, unlike AR cells, photoheterotrophically grown PCC‐M cells formed non‐easily‐breakable aggregates, and reliable measurement of PCC‐M cell density was not possible. Full‐length HlyA protein is present in all GT stains (Fig. 4) and, unlike PCC‐M cells (truncated HlyA protein), they do not show a clumping phenotype in the presence of glucose. HlyA has been connected to glucose tolerance earlier as HlyA‐less cells do not grow mixotrophically at low light [45]. Thus, it is tempting to speculate that the HlyA protein plays a role in glucose tolerance. The PCC‐M chromosome contains an SNP in −56 position upstream of the *glcP* glucose transporter gene, but it remains to be studied if that mutation plays a role in glucose tolerance. We suggest that glucose tolerances in GT and PCC‐M strains are of different origins.

Construction of the phylogenetic tree of different substrains is complicated by some unexpected sequence variation. First of all, strains originating from Vermaas's laboratory do not share common mutations (Fig. 4). Furthermore, the 18 bp deletion upstream of the *rpoZ* gene found in all PCC strains is also present in GT‐I strain but not in any other GT strains. The consequences of the reduced amounts of the ω subunit in PCC and GT‐I strains remain to be studied. The ω less ΔrpoZ strain is fully viable in standard growth conditions [19], but ΔrpoZ cells do not acclimate to high CO_2_ [55] or high temperature [56]. And finally, the *rpl3* gene contains the same mutation in GT‐W and GT‐P but not in the other GT substrains (Fig. 4). As *Synechocystis* contains many copies of the genome, one possible explanation is that all copies are not completely identical, and depending on growth conditions, different chromosome variants become the prevailing forms, and only those most frequent variants are considered and published as the outcomes of the genome sequencing projects.

Obviously, all laboratory substrains contain unique mutations causing phenotypic differences between so‐called ‘wild‐type’ strains. Comparison of results from different laboratories and selection of an appropriate substrain for each study would be easier after sequencing all ‘wild‐type’ substrains. For example, our GT‐T substrain or mutants constructed using it as a host strain are not compatible with studies involving mixotrophic or heterotrophic conditions using glucose as a carbon source because the frameshift mutation in the glucose transporter prevents utilization of externally added glucose.

## Conflict of interest

The authors declare no conflict of interest.

## Author contributions

TT designed and supervised this work; SK performed the data analyses; JK isolated DNA and measured growth, chlorophyll, and RNAP and Rubisco subunit contents of cells; and ML performed pilus staining. SK and TT wrote the manuscript with contributions from JK and ML All authors have read and agreed to the published version of the manuscript.

## Data accessibility section

The genome sequence has been deposited at NCBI under GenBank accession CP094998 https://www.ncbi.nlm.nih.gov/nuccore/CP094998 and BioProject accession number PRJNA821690 https://www.ncbi.nlm.nih.gov/bioproject/PRJNA821690, BioSample accession number SAMN27121261 https://www.ncbi.nlm.nih.gov/biosample/SAMN27121261, and SRA number SRR18670503 https://www.ncbi.nlm.nih.gov/sra/PRJNA821690.
